# Isolated Splenic Metastasis of Primary Lung Cancer Presented as Metachronous Oligometastatic Disease—A Case Report

**DOI:** 10.3390/diagnostics12010209

**Published:** 2022-01-15

**Authors:** Milorad Reljic, Boris Tadic, Katarina Stosic, Milica Mitrovic, Nikola Grubor, Stefan Kmezic, Miljan Ceranic, Vladimir Milosavljevic

**Affiliations:** 1 Department for HBP Surgery, Clinic for Digestive Surgery, University Clinical Centre of Serbia, Koste Todorovica Street, No. 6, 11000 Belgrade, Serbia; misoreljic@gmail.com (M.R.); n.grubor@yahoo.com (N.G.); kstefan1986@gmail.com (S.K.); miljanceranic1972@gmail.com (M.C.); 2 Department for Surgery with Anesthesiology, Faculty of Medicine, University of Belgrade, Dr Subotica No. 8, 11000 Belgrade, Serbia; 3 Center for Radiology and Magnetic Resonance Imaging, University Clinical Centre of Serbia, Pasterova No.2, 11000 Belgrade, Serbia; katestosic@gmail.com (K.S.); dr_milica@yahoo.com (M.M.); 4 Department for HBP Surgery, University Hospital Medical Center Bezanijska kosa, Dr Zorza Matea bb, 11000 Belgrade, Serbia; milosavljevicvladimir10@gmail.com

**Keywords:** isolated splenic metastasis, lung cancer, oligometastatic disease

## Abstract

Modern oncology practice and new antitumor drugs prolonged disease-free intervals in patients with lung cancer. Patients with distant metastatic disease are treated only with palliative intent. The International Association for the Study of Lung Cancer, in the 8th edition of the TNM classification, for the first time includes oligometastatic disease as a clinical state that describes the patients with distant metastasis, limited in number and organ sites, who may have more indolent biology. In this paper, we present a case of a 56-year-old man who was admitted to our clinic regarding a radiologically diagnosed splenic lesion of uncertain nature, and who underwent a left upper lobectomy for primary lung cancer 12 years before. After a detailed radiological diagnosis, it was concluded that it is highly suspected metastatic lesion of the spleen and the patient underwent a splenectomy. While no definitive protocols exist on the management of isolated splenic metastasis from lung cancer, splenectomy, in suitable patients, with reasonable survival expectations, improves patient disease-free survival and can prevent potentially life-threatening complications, such as splenic rupture. ^18^F-FDG PET has very high sensitivity and specificity for differentiating benign and malignant splenic lesions especially in patients who are in the follow up protocol due to primary malignancy.

## 1. Introduction

Splenic metastatic disease is a rare clinical entity with a total prevalence of 2.3–7.1% [1]. However, isolated metastases in the spleen (ISM), of non-hematological solid malignancy origin, occur only exceptionally, in 0.6–1.1% of cases [2]. The primary origin of these tumors is most commonly melanoma, colorectal cancer, breast cancer, and ovarian cancer [3]. It is assumed that secondary lesions in the spleen are most likely caused by hematogenous dissemination, since there are no lymphatic afferents [4].

Lung carcinoma is a highly metastatic disease that has some preferential sites for metastasis, such as the brain, bones, and adrenal glands [5]. These patients are treated mainly with palliative intent [6]. Lung cancer rarely metastasizes to the spleen without the involvement of the other organs. Surgery has an important role in the management of such patients because survival is favorable in many cases.

In this paper, we present a case of a 56-year-old man who was admitted to our clinic regarding a radiologically diagnosed splenic lesion of uncertain nature, and who underwent a left upper lobectomy for primary lung cancer 12 years before.

## 2. Case Report

A 56-year-old patient was referred from the oncology council of the Clinic for Pulmonology of the University Clinical Center of Serbia on 1 December 2020 for additional evaluation of a splenic lesion of uncertain nature. The patient has been scheduled for oncological follow-up appointments for several years since, 12 years before, he underwent surgery for lung cancer, when a left upper lobectomy was performed. Histological examinations confirmed small cell lung cancer (T1N3M0). After surgery, the patient received adjuvant chemo- and radiotherapy (Carboplatin and Paclitaxel). A few days before the council, an abdominal ultrasound examination performed in an outdoor hospital revealed a large tumorous mass in the middle of the spleen.

**Figure 1:**
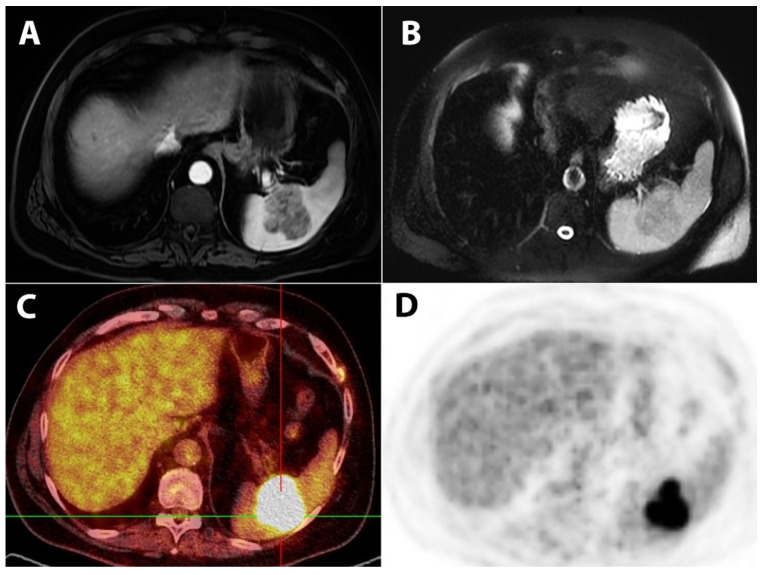
MRI T1-weighted FS image after gadolinium enhancement reveals a predominantly hypointense lobulated, relatively homogeneous splenic lesion (**A**) with discrete low signal intensity on T2W. (**B**) FDG-PET/CT (**C**) and FDG-PET axial image (**D**) show a splenic lesion with intensely increased FDG uptake.

Due to the precise differentiation of the lesion, additional diagnostic methods were performed upon admission of the patient to our clinic. Abdominal magnetic resonance imaging (MRI) revealed a solitary well-circumscribed lobulated solid lesion in the spleen that was mildly hypointense on the T2-weighted imaging, isointense on the T1-weighted imaging, and showed lower enhancement after intravenous contrast administration (Figure 1A,B). Considering the MRI finding, the patient was referred to positron-emission tomography (PET) using 18F-fluorodeoxyglucose (FDG) as a radiotracer. This exam confirmed that the splenic tumor shows increased metabolic activity, so the change was characterized as a highly suspected metastatic lesion (Figure 1C,D).

**Figure 2:**
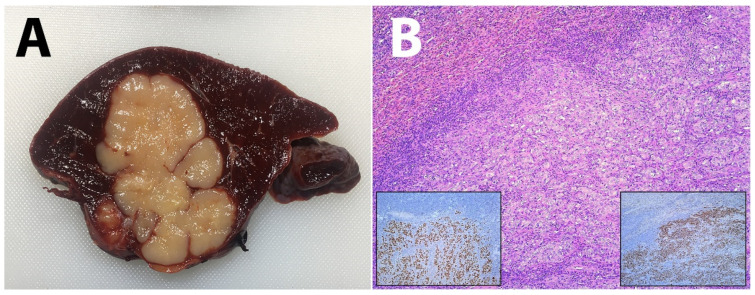
Macroscopic appearance of metastatic change on the cross-section of the spleen (**A**). Poorly differentiated carcinoma, showing on immunohistochemical examination strong nuclear immunoexpression of TTF-1 and cytoplasmic immunostaining of napsin A (**B**).

A splenectomy was performed. The resected spleen weighed 320 g; the tumor was 60 × 55 × 40 mm in diameter, its boundary with the surrounding tissue was well defined, and the cut surface was yellowish white (Figure 2A). The splenic tumor histologically revealed poorly differentiated carcinoma, showing on immunohistochemical examination strong nuclear immunoexpression of TTF-1 and cytoplasmic immunostaining of napsin A, findings consistent with metastatic pulmonary adenocarcinoma (Figure 2B).

Besides the reactive thrombocytosis, the postoperative period was uneventful. On the fifth postoperative day, the patient was discharged from the hospital. Immunization against Hemophilus influenzae, pneumococci, and meningococci were prescribed in order to prevent post-splenectomy infection complications. Patient was referred back to the oncology council. Three cycles of platinum/etoposide-containing regimen chemotherapy were prescribed.

## 3. Discussion

Lung cancer with isolated splenic metastasis is exceedingly rare. Searching the bibliographic databases (Pubmed, Scopus, Web of Science), ISM of primary lung cancer has been reported only in 34 cases (Table 1). The secondary splenic lesion is mostly seen in the diffuse disease, when widespread hematologic dissemination usually involves 4–5 other organs, with an incidence of 1.2–5.6% [7,8].

Several hypotheses might explain why spleen metastasis is a fairly rare occurrence. A constant flow of splenic sinus blood may reduce cancer cell adhesion to the spleen [9]. According to Sappington, a low incidence of splenic metastasis is explained by a sharp angle of the splenic artery with a celiac axis [10]. Kettle suggested that contraction of smooth muscle within the splenic capsule might prevent the growth of tumor emboli [11]. Splenic microenvironment humoral factor that destroys cancer cells and avoids their adhesion and pronounced phagocytic activity has also been suggested as possible factors preventing malignant cell development in the spleen [12,13].

Analyzing the previous 34 cases from Table 1, one half (50%) of ISM were detected simultaneously with a diagnosis of lung cancer. However, it could be found metachronously, long after the primary lesions were diagnosed. In most cases, a splenic lesion was detected up to 2 years after the diagnosis of the primary tumor. The longest lag period was reported in a 49-year-old man with a history of right lobectomy for a carcinoid tumor 8 years before [14]. However, in our patient, the splenic metastasis was detected 12 years after the surgery for lung adenocarcinoma.

Although some previous articles indicate that metastases in the spleen are more common in left lung cancer, Table 1 shows that ISM occurs equally regardless of which lung is affected by the primary disease [9].

In most cases, splenic metastasis has a clinically silent course. Some of the patients have complaints of abdominal pain, discomfort, bloating or fever, but most of them are asymptomatic. As ISM rarely manifests clinical symptoms, it is usually found by coincidence as an incidental finding during follow-up imaging diagnostic or as a part of an evaluation for different diseases. There are some reports of nontraumatic splenic rupture on the grounds of ISM, with profuse intraabdominal bleeding [15,16,17]. In such a life-threatening condition that can be present with acute abdominal symptoms, urgent splenectomy is indicated as a life-saving procedure.

The majority of splenic metastases are identified by ultrasonography or CT scan, knowing that most of the solitary splenic metastases are asymptomatic. In the case of finding isolated splenic lesion > 1 cm during the oncological follow-up, splenic metastases should be suspected. Splenic metastases can have different presentations on computed tomography (CT) and magnetic resonance imaging (MRI), ranging from cystic to solid lesions and showing various enhancement models. Imaging features that favor metastases are heterogeneity, poorly defined margins, and multiplicity and, in the case of presence of these imaging characteristics, further evaluation is needed. 18F-FDG PET has very high sensitivity and specificity for differentiating benign and malignant splenic lesions, especially in patients who are in the follow-up protocol due to primary malignancy [18]. It is not always easy to reveal the nature of splenic change seen on conventional radiological examination. Given that the patient had or currently has lung cancer, the malignancy must be ruled out. A splenic biopsy is considered a valuable diagnostic method for differentiating benign from malign lesions [19,20]. Apart from possible splenic rupture, bleeding, and peritoneal dissemination a splenic biopsy may not be reliable [21,22]. Therefore, we firmly believe that having a preoperative pathohistological finding is not going to change the corresponding treatment or the surgical outcome. Splenectomy, either open or laparoscopic, is at the same time the best diagnostic and therapeutic method [23].

Traditionally, patients with metastatic lung cancer have been managed with chemotherapy and palliative treatments aimed only to prolong and improve quality of life and relieve symptoms. Although only one site of recurrence or metastasis is present, the tumor cells can be disseminated throughout the body hematogenously, meaning that local therapy cannot eradicate all cancer cells. The International Association for the Study of Lung Cancer, in the 8th edition of the TNM classification, for the first time includes oligometastatic disease [24]. Oligometastasis is a clinical state that describes the patients with distant metastasis, limited in number and organ sites, who may have more indolent biology [25]. Considering the therapeutic principle of oligometastatic disease for solitary brain or adrenal metastasis, splenectomy should be offered as a therapeutic option for these patients.

The optimal surveillance of patients for recurrence after surgical resection of lung cancer is controversial. Wide variations in follow-up modalities are observed worldwide. Distant metastatic disease is considered incurable and mainly treated when symptomatic, so active extrathoracic surveillance to detect asymptomatic metastatic disease is not warranted [26]. Since that splenic metastatic disease is generally asymptomatic, but with a good chance of treatment success, periodic abdominal ultrasound examinations could be useful during patient follow-up.

## 4. Conclusions

Modern oncology practice and new antitumor drugs prolong disease-free intervals in patients with lung cancer. While no definitive protocols exist on the management of isolated splenic metastases, splenectomy, in suitable patients, with reasonable survival expectations, improves patient disease-free survival and can prevent potentially life-threatening complications, such as splenic rupture. In our opinion, given all the benefits of minimally invasive surgery, laparoscopic splenectomy should be the therapy of choice.

## Figures and Tables

**Table 1:** Characteristics of patients who developed isolated splenic metastasis from lung carcinoma.

No.	First Author/Year	Histology (Primary Lung Lesion)	Lung Lesion Side	Time to Splenic Metastasis	Sex	Age	Metastasis Symptoms	Treatment of Primary Tumor	Treatment of Splenic Metastasis	Follow-up at the Time of the Report
1.	Klein/1987 [12]	Bronchioalveolar carcinoma	Right	20 months	F	57	Abdominal pain	Right lower and middle lobectomy	Splenectomy	Died 49 months after splenectomy
2.	Edelman/1990 [27]	Poorly differentiated adenocarcinoma	Left	0 months	F	63	Asymptomatic	n.a	n.a	n.a
3.	Macheers/1992 [28]	Large-cell undifferentiated carcinoma	Left	0 months		n.a.	Asymptomatic	n.a.	Splenectomy	Died 1 month after splenectomy
4.	Gupta/1993 [15]	Squamous cell carcinoma	Right	0 months		n.a.	Splenic rupture	n.a.	Splenectomy	Died 8 weeks after splenectomy
5.	Kinoshita/1995 [9]	Squamous cell carcinoma	Left	14 months	M	72	Asymptomatic	Surgical removal of primary tumor	Splenectomy	Died 27 months after splenectomy
6.	Takada/1998 [14]	Bronchopulmonary carcinoid tumor	Right	96 months	M	49	Abdominal pain	Right upper lobectomy	Splenectomy	Disease free after 8 years
7.	Tomaszewski/2003 [29]	Lung cancer	Left	0 months	M	68	Asymptomatic	Upper left lobectomy	Splenectomy	n.a.
8.	Massarweh/2001 [16]	Poorly differentiated adenocarcinoma	Left	0 months	M	68	Splenic rupture	Palliative chemotherapy	Splenectomy	n.a.
9.	Schmidt/2004 [30]	Moderately differentiated adenocarcinoma	Left	25 months	M	72	Asymptomatic	Surgical removal of primary tumor	n.a.	Disease free after 2 years
10.	Pramesh/2004 [31]	Squamous cell carcinoma	Left	2 months	M	55	Asymptomatic	Combined radiochemotherapy	chemotherapy	n.a.
11.	Lachachi/2004 [17]	Poorly differentiated carcinoma	Right	0 months		n.a.	Splenic rupture	n.a.	Splenectomy	n.a.
12.	Sánchez-Romor/2006 [32]	Adenocarcinoma	Left	0 months	M	73	Abdominal pain	Left lung resection	Splenectomy	n.a.
13.	Van Hul/2008 [33]	Adenocarcinoma	Left	24 months	M	67	Asymptomatic	Surgical removal of primary tumor	Splenectomy	n.a.
14.	Ando/2009 [34]	Squamous cellcarcinma	Right	10 months	M	71	Asymptomatic	Combined radiochemotherapy	Splenectomy	n.a.
15.	Chloros/2009 [35]	Squamous cellcarcinma	Right	0 months	M	59	Asymptomatic	Surgical removal of primary tumor	Splenectomy	n.a.
16.	Tang/2010 [36]	Large-cell undifferentiated carcinoma	Right	4 months	F	49	Fever	Lobectomy of the right middle and lower lobe	Splenectomy	n.a.
17.	Scintu/1991 [37]	Large-cell anaplastic carcinoma	n.a.	0 months		n.a.	Asymptomatic	Pulmonary lobectomy	Splenectomy	Disease free after 41 months
18.	Yen/2005 [38]	Adenocarcinoma	Left	24 months	M	56	Asymptomatic	Left pneumonectomy	Splenectomy	n.a.
19.	Fujii/2008 [39]	Poorly differentiated adenocarcinoma	Left	3 months	M	58	Asymptomatic	Left upper lobectomy	Splenectomy	n.a.
20.	Assouline/2006 [40]	Large-cell undifferentiated carcinoma	Right	21 months	M	77	Abdominal pain	Right pneumonectomy	Splenectomy	Disease free after 2 years
21.	Eisa/2014 [41]	Adenocarcinoma	Right	0 months	F	53	Abdominal pain	Surgical removal of primary tumor	Splenectomy	Disease free at the time of the report
22.	Belli/2016 [42]	Large-cell carcinoma	Right	60 months	M	65	Asymptomatic	Right pneumonectomy	n.a.	n.a.
23.	Sardenberg/2013 [43]	Adenocarcinoma	Right	7 months	F	49	Abdominal pain	Right upper lobectomy	Splenectomy	Disease free after 96 months
24.	Dias/2012 [44]	Squamous cell carcinoma	Right	16 months	M	82	Asymptomatic	Right bilobectomy	Splenectomy	Disease free after 12 months
25.	Cai/2015 [45]	Adenocarcinoma	Right	17 months	F	56	Asymptomatic	Right lower lobectomy	Splenectomy	n.a.
26.	Soussan/2011 [46]	Adenocarcinoma	n.a.	0 months	M	52	Asymptomatic	n.a.	n.a.	n.a.
27.	Iguchi/2015 [47]	Adenocarcinoma	Left	12 months	F	63	Asymptomatic	Left lower lobectomy	Splenectomy	n.a.
28.	Mitsimponas/[48]	Adenocarcinoma	Right	0 months	F	66	Asymptomatic	Radiochemotherapy	Chemotherapy	Alive at the time of the report
29.	Hara/2017 [49]	Poorly differentiate adenocarcinoma	Right	0 months	F	81	Asymptomatic	Right upper lobactomy	Lap. splenectomy	n.a.
30.	Zeng/2018 [50]	Adenoid cystic carcinoma	Right	48 months	F	38	Abdominal pain	Right middle lobectomy	Splenectomy	n.a
31.	Lopera/2018 [51]	Large cell carcinoma	Right	n.a.	F	69	Abdominal pain	Right upper lobactomy	Lap. Splenectomy	n.a
32.	Tanaka/2020 [52]	Squamous cell carcinoma	Righ	0 months	M	78	Abdominal pain	Surgery	Splenectomy	n.a.
33.	Ousama/2001 [53]	Non-small-cell lung cancer	Left	0 months	M	58	Abdominal pain	Chemotherapy	Splenectomy	n.a.
34.	Grant/2020 [54]	Adenocarcinoma	Right	n.a.	F	73	Asymptomatic	Right lower lobe lobectomy	Splenectomy	Alive at the time of the report
35.	Present case	Adenosquamos carcinoma	Left	144 months	M	56	Asymptomatic	Left upper lobectomy	Splenectomy	Disease free after 24 months
